# Yao syndrome: Report of 3 cases with a family history

**DOI:** 10.1016/j.jdcr.2026.05.058

**Published:** 2026-06-03

**Authors:** Miran Rezhan, Galen T. Foulke, Matthew F. Helm

**Affiliations:** a Pennsylvania State College of Medicine, Hershey, Pennsylvania; b Department of Dermatology, Penn State Hershey Medical Center, Hershey, Pennsylvania; c Department of Public Health Sciences, Penn State Hershey Medical Center, Hershey, Pennsylvania

**Keywords:** autoinflammatory, canakinumab, case series, dermatitis, NOD2, Yao syndrome

## Introduction

Yao syndrome (YAOS) is a systemic autoinflammatory disease (AID) characterized by periods of fevers, dermatitis, polyarthritis, and gastrointestinal (GI) and sicca-like symptoms associated with specific nucleotide-binding oligomerization domain (NOD)-containing 2 variants.^1^ *NOD2* variants have been associated with Crohn disease (CD) and Blau syndrome (BS) in addition to YAOS.^2^^,^^3^ The NOD2 protein, in tandem with the NOD1 protein, plays a crucial role in innate immunity through its ability as an intracellular cytosolic receptor for pathogen-associated molecular patterns. YAOS was initially identified as a novel AID that resembled BS and CD, but with key differences. Among these differences are the increased cases of nongranulomatous dermatitis seen in YAOS compared with BS and CD.^4^ YAOS has a prevalence of 1 to 10/100,000 and a predominance in the female population, which underscores its rarity. YAOS is not inherited in a simple Mendelian way but is linked to variations in the *NOD2* gene. Most patients carry 2 or more gene variants, suggesting a “2-hit model” where combinations of mutations increase risk.^4^ Even with *NOD2* mutations, many people never develop the syndrome, highlighting the role of other genetic or environmental factors. Current treatment strategies involve the use of sulfasalazine, glucocorticoids, colchicine, and anti-tumor necrosis factor-α agents, conventional antirheumatic drugs such as hydroxychloroquine, and biologics. The most used biologics are canakinumab, an interleukin (IL) 1β inhibitor, and anakinra, an IL-1 receptor antagonist.^5^ In this work, we report the effectiveness of canakinumab use in a case of severe YAOS, and the importance of symptom-specific treatment in milder phenotypes.

### Patient 1

A family of a mother, daughter, and son presented to dermatology with varying symptoms in relation to confirmed *NOD2* mutations. The first patient is the 17-year-old daughter who presented to dermatology for an assessment of potential hidradenitis suppurativa and for the possibility of YAOS or BS. She had reported low-grade fevers up to 100.8 °F, arthritis, fatigue, and malaise. Additionally, she reported rashes that worsened in the heat, along with tender nodules in her armpits. The patient had a past medical history of Ehlers-Danlos syndrome, postural orthostatic tachycardia syndrome, tethered cord (s/p surgery), and mast cell activation syndrome.

**Fig 1:**
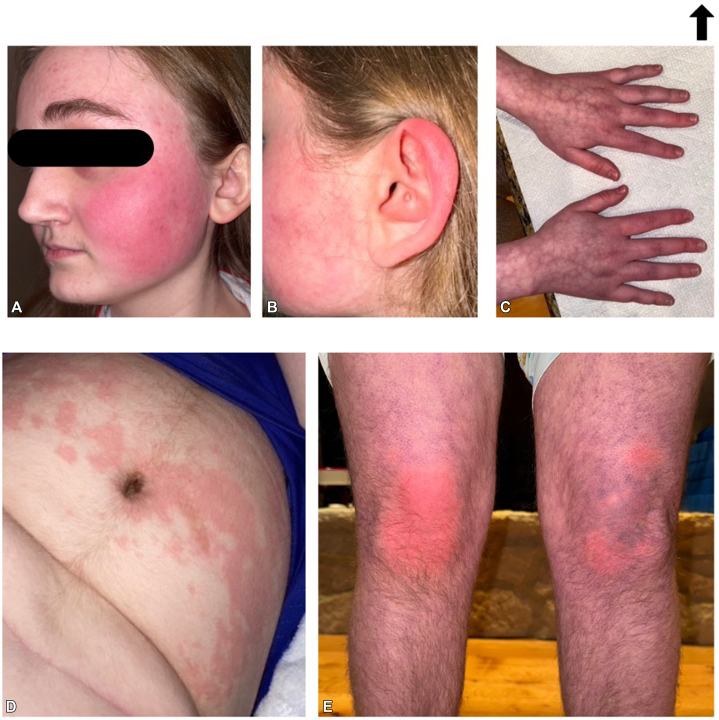
Yao syndrome: **(A)** erythematous patches on the malar cheeks, **(B)** pink helices of the ears, **(C)** prominent livedo reticularis on the wrists and hands, **(D)** pink migratory patches on the abdomen, and **(E)** salmon-colored patches on the knees.

On physical examination, she presented with flushed cheeks (Fig. 1, *A*), livedo reticularis of the arms and lower legs (Fig 1, *C*), and follicular prominence of the arms and legs. Symptoms of dermatitis were also described by the patient, with an eczematous rash being noted on the physical examination as well. Photographs were provided of migratory pink urticarial plaques (Fig 1, *D* and *E*), along with intermittently red, swollen eyelids. Upon performing a Periodic Fever 6-gene next-generation Sequencing panel that included *MEFV, MVK, NLRP3, TNFRSF1A, LPIN2,* and *PSTPIP1*, a heterozygous *NOD2* IVS8^+158^ variant and a heterozygous *NOD2* p.Val972Ile variant were identified in the patient with no other abnormal findings.^1^ Ultimately, she reported more than 2 episodes of skin and fever flares, arthritic symptoms in multiple joints in combination with a rare variant *NOD2* mutation in the absence of any exclusionary criteria for YAOS, supporting a YAOS diagnosis. We did not perform a biopsy of the rashes, as on histopathology YAOS typically presents with nonspecific spongiotic dermatitis. Therefore, the diagnosis was made by the clinical and diagnostic criteria discussed.

She was diagnosed with YAOS in the setting of her dermatologic and systemic findings along with her *NOD2* mutation. Prior treatments attempted were doxycycline 50 mg twice a day and topical clindamycin to regulate her skin symptoms. Because of the severity of her symptoms, low-grade fevers, and coexistent hidradenitis suppurativa, she was started on 37.5 mg of canakinumab subcutaneously, which triggered mast cell symptoms, prompting termination of the treatment. Premedication was then recommended, which was hydrocortisone given the day, the hour before, and for the 2 days following injection. She was started again on canakinumab at a dosage of 37.5 mg, then reached the recommended dose of 150 mg over the course of 4 total injections every 4 weeks.

The premedication made the previous mast cell symptoms following injection tolerable. The injections also caused some vertigo-like symptoms but were manageable. Following premedication and treatment with canakinumab, her fever, rash, and headaches improved.

### Patient 2

**Fig 2:**
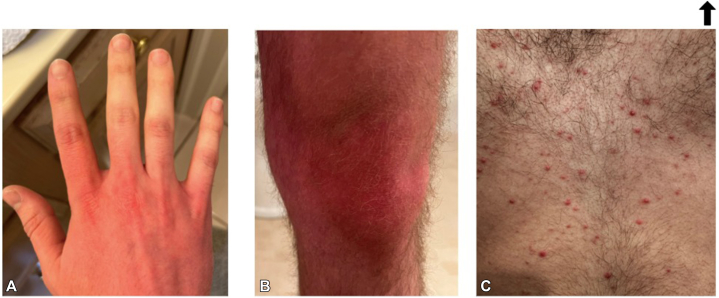
Yao syndrome: **(A)** Raynaud phenomenon, **(B)** pink patches on the knee, and **(C)** red follicular-based papules.

The second patient is patient 1’s 21-year-old brother who presented to dermatology with GI issues, pericarditis, and periodic fevers as high as 103 °F that were accompanied by a rash. The patient had a past medical history of Ehlers-Danlos syndrome and postural orthostatic tachycardia syndrome. Following genetic screening, the only abnormal finding was a heterozygous *NOD2* p.Val972Ile variant with features of CD along with a family history of YAOS. On physical examination, he had pink papules and pustules on the back and around the mouth (Fig 2, *C*), papules on the arms, and pictures showed macules and patches on the hands and knees (Fig 2, *B*).

The patient was diagnosed with CD by GI-based colonoscopy. He was not diagnosed with YAOS due to his CD diagnosis, an exclusion criterion for YAOS diagnosis.^1^ The patient used colchicine and doxycycline previously to control the periodic fevers and rash unsuccessfully. He then experienced severe pericarditis requiring hospitalization and was prescribed rilonacept to be administered once a week subcutaneously at a dose of 160 mg. After rilonacept initiation, his pericarditis improved, and maximum fever decreased to 99.5 °F. Risankizumab was recommended to address his GI symptoms, and following administration, he reported improvement of those symptoms. In this case, medication was prescribed for symptom mitigation, rather than for a specific NOD2 disease diagnosis.

### Patient 3

The third patient is the 51-year-old mother of patients 1 and 2, who presented to dermatology with multiple episodes of mild GI-related symptoms and migratory burning and itchy rashes along with an *NOD2* mutation. After genetic screening was performed, a heterozygous *NOD2* p.Val972Ile variant was the only abnormal finding identified. Although this variant alone was not stated outright in the molecular criterion for YAOS, it was interpreted as a rare variant in the presence of her symptoms.^1^ She stated having joint pain and livedo reticularis but denied periodic fevers. The patient had a past medical history of Ehlers-Danlos syndrome, dysautonomia, and Raynaud’s syndrome.

**Fig 3:**
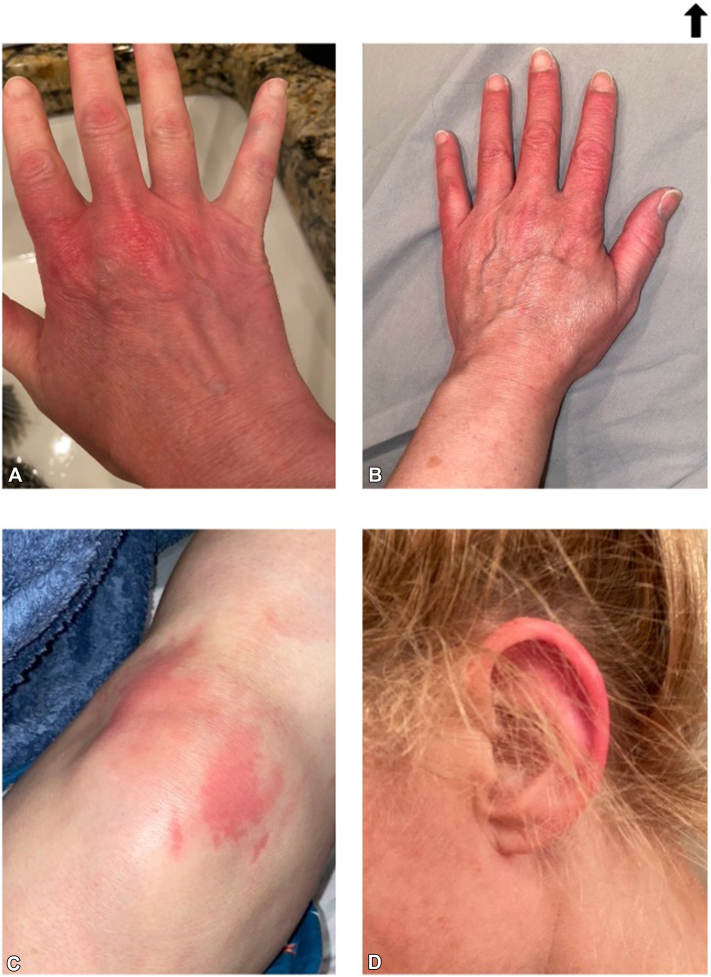
Erythromelalgia: **(A** and **B)** pink dorsal hands with swollen digits, consistent with erythromelalgia, **(C)** migratory pink patches on the knees, and **(D)** pink helices.

On physical examination, pink papules on the arms and legs, and pictures provided by the patient showed bright red swollen hands indicative of erythromelalgia (Fig 3, *A* and *B*), and tan patches on the chest were seen. Additionally, symptoms of dermatitis were described by the patient in the past, and eczematous rash was also noted on physical examination. A skin biopsy was not performed on her rashes because of the non-specificity of YAOS histopathology. Therefore, because she presented with 2 or more episodes of dermatitis symptoms, GI symptoms, and a rare *NOD2* gene variant, a diagnosis of YAOS was made.

Following diagnosis, the patient was started on hydroxychloroquine 400 mg tablets daily. However, she was unable to tolerate the metallic taste and burning of her throat, which she had also experienced previously with colchicine. She used amitriptyline/gabapentin/lidocaine cream for her erythromelalgia effectively. Treatment focused on mitigating symptoms rather than use of biologics or glucocorticoids for YAOS.

## Discussion

The pathophysiology of YAOS is still incompletely understood, but it is thought that it involves proinflammatory cytokines and the hyperactivation of NOD2-mediated signaling pathways.^6^ The cases presented in this work demonstrated the promise surrounding the use of canakinumab in YAOS. This is consistent with previous reports of effective canakinumab use in inflammatory conditions that anakinra has been unable to address. It has been postulated that the therapeutic benefit of canakinumab use over anakinra is the longer duration of action that it possesses.^7^ Canakinumab blocks IL-1β activity, which stops IL-1β and the IL-1 receptor interactions, thus stopping subsequent inflammatory reactions.^8^

In large cohorts, approximately 65% to 70% of patients with YAOS respond to IL-1 inhibition, supporting its use in systemic or refractory disease.^4^ IL-6 inhibitors may be considered in refractory cases, although reported response rates are lower.^4^^,^^9^ Hydroxychloroquine and sulfasalazine are commonly used first-line options in milder disease because of their safety, cost, and steroid-sparing potential, whereas colchicine appears to have limited efficacy.^4^^,^^5^

YAOS is characterized by periods of fevers, dermatitis, polyarthritis, and GI and sicca-like symptoms. The most documented cutaneous symptoms of YAOS have been erythematous plaques and patches. On pathology, key findings have included a combination of both lymphocytic and neutrophilic infiltrates, granulomatous findings, as well as spongiotic dermatitis.^10^^,^^11^ In the cases described in this series, erythematous patches were seen in all 3 patients. Additionally, recent literature has uncovered other skin symptoms such as livedo, petechial rashes, and facial flushing.^10^ The livedo and facial flushing patterns were highlighted well in the daughter in this case series. On histopathology, YAOS typically presents with a spongiotic dermatitis, which is very nonspecific, making the diagnosis primarily clinical.^11^ Overall, although YAOS is associated with the classic constellation of symptoms as described above, our patients had some variability in their presentation, namely with livedo, petechial rashes, and facial flushing that has only been more recently characterized in patients with YAOS.

A clinical diagnosis of YAOS requires 2 major criteria, at least 1 minor criterion, the molecular criterion, and exclusion criteria. The major criteria include periodic occurrence of flares (≥2 episodes) and recurrent fever and/or dermatitis. The 4 minor criteria include oligo- or polyarthralgia, inflammatory arthritis, or distal extremity swelling; abdominal pain or diarrhea; sicca-like symptoms; and pericarditis or pleuritis. The molecular criterion is *NOD2* IVS8^+158^ or R702W or both, or other rare variants. The exclusion criteria include high titer antinuclear antibodies; inflammatory bowel disease; BS; adult sarcoidosis; primary Sjögren syndrome; and monogenic AIDs.^1^ The mother and daughter in this case series were thus diagnosed with YAOS, whereas the son was diagnosed with CD previously, excluding a YAOS diagnosis. However, the son was also genetically screened and had the same *NOD2* genetic variation as his mother and sister; thus, we thought it was relevant to comment on his clinical presentation as well.

YAOS has been reclassified as a genetically transitional disease rather than a monogenic disorder, meaning that genetic variation may contribute to disease risk without being sufficient to cause disease.^12^ This framework aligns with ClinGen recommendations for low-penetrance variants and risk alleles.^13^ A recent All of Us case-control study confirmed increased prevalence of *NOD2* IVS8^+158^ in YAOS and showed coinheritance of IVS8^+158^ with R702W or L1007fs, supporting the inclusion of these variants in YAOS diagnostic criteria.^14^

Literature regarding the impact of the heterozygous *NOD2* p.Val972Ile variant on manifestation of YAOS is limited. In this case series, all 3 patients had this genotype, with varying clinical presentations. Both the mother and son had the *NOD2* p.Val972Ile variant in the absence of other *NOD2* variants, yet the mother was diagnosed with YAOS, whereas the son was diagnosed with CD, excluding a YAOS diagnosis. On the other hand, the daughter was found to have both the *NOD2* p.Val972Ile variant as well as the *NOD2* IVS8^+158^ variant that is more commonly associated with patients with YAOS in previous work.^14^ Shared NOD2 p.Val972Ile variants were observed across family members; however, given the genetically complex nature of YAOS, this case series cannot determine a definitive inheritance pattern or familial segregation of disease.

YAOS is a highly variable and heterogeneous condition with variable symptoms. This case series highlights this nicely even within the same family. In the first case, canakinumab was given to improve the traditional symptoms of YAOS such as episodes of fevers, rashes, and headaches. In the second case, treatment was dictated by the symptoms, such as rilonacept for pericarditis and fever or risankizumab for the GI symptoms brought on by CD. In the third case, amitriptyline/gabapentin/lidocaine cream was effective for reducing the skin irritation noted by the patient.

## Conclusion

YAOS is a genetically complex AID associated with NOD2 variants and variable dermatologic and systemic manifestations. These 3 related cases demonstrate phenotypic variability among patients with shared NOD2 p.Val972Ile variants, while also emphasizing that genotype alone is insufficient to establish disease. Management should be guided by clinical phenotype, disease severity, and dominant symptom burden.

## Conflicts of interest

None disclosed.
